# Structure and function of the cytochrome P450 peroxygenase enzymes

**DOI:** 10.1042/BST20170218

**Published:** 2018-02-06

**Authors:** Andrew W. Munro, Kirsty J. McLean, Job L. Grant, Thomas M. Makris

**Affiliations:** 1 Manchester Institute of Biotechnology, School of Chemistry, The University of Manchester, 131 Princess Street, Manchester M1 7DN, U.K.; 2 Department of Chemistry and Biochemistry, University of South Carolina, Columbia, SC 29208, U.S.A.

**Keywords:** alkenes, cytochrome P450, enzyme mechanism, iron-oxo species, peroxygenase, substrate decarboxylation

## Abstract

The cytochromes P450 (P450s or CYPs) constitute a large heme enzyme superfamily, members of which catalyze the oxidative transformation of a wide range of organic substrates, and whose functions are crucial to xenobiotic metabolism and steroid transformation in humans and other organisms. The P450 peroxygenases are a subgroup of the P450s that have evolved in microbes to catalyze the oxidative metabolism of fatty acids, using hydrogen peroxide as an oxidant rather than NAD(P)H-driven redox partner systems typical of the vast majority of other characterized P450 enzymes. Early members of the peroxygenase (CYP152) family were shown to catalyze hydroxylation at the α and β carbons of medium-to-long-chain fatty acids. However, more recent studies on other CYP152 family P450s revealed the ability to oxidatively decarboxylate fatty acids, generating terminal alkenes with potential applications as drop-in biofuels. Other research has revealed their capacity to decarboxylate and to desaturate hydroxylated fatty acids to form novel products. Structural data have revealed a common active site motif for the binding of the substrate carboxylate group in the peroxygenases, and mechanistic and transient kinetic analyses have demonstrated the formation of reactive iron-oxo species (compounds I and II) that are ultimately responsible for hydroxylation and decarboxylation of fatty acids, respectively. This short review will focus on the biochemical properties of the P450 peroxygenases and on their biotechnological applications with respect to production of volatile alkenes as biofuels, as well as other fine chemicals.

## The cytochromes P450 — a brief introduction

The cytochromes P450 (P450s, CYPs) were the first group of enzymes to be classified as a ‘superfamily’ through bioinformatics studies performed by Nebert et al. [1], and the number of P450s classified into the P450 superfamily had extended to more than 21 000 members by August 2013 [2]. P450s are heme *b*-binding enzymes in which the heme iron is typically ferric in the resting, purified state, but can be ferrous *in vivo* [3]. The heme iron is proximally co-ordinated by a cysteine residue in its thiolate (deprotonated) form, and this ligation state is considered to an active form of the enzyme. The cysteine thiolate-co-ordinated ferric P450 typically has its major (Soret band) absorbance maximum at ∼418 nm [4]. In its ferrous state, it binds to the inhibitor carbon monoxide (CO) to give a Fe^II^–CO complex in which the major (Soret) absorbance band lies at ∼450 nm — i.e. pigment (with absorption peak at 450 nm) [5,6]. This spectral shift provides the basis for the naming of these enzymes and reflects the retention of cysteine thiolate proximal co-ordination in this form of the enzyme. Protonation of the thiolate results in a Fe^II^–CO complex with Soret absorbance maximum at ∼420 nm, and this thiol co-ordinated state is often considered to be an inactivated form of the enzyme [7]. However, recent studies have shown that the P450 form can sometimes be recovered from P420 by the addition of substrate or alteration of pH [8–10].

**Figure 1.**
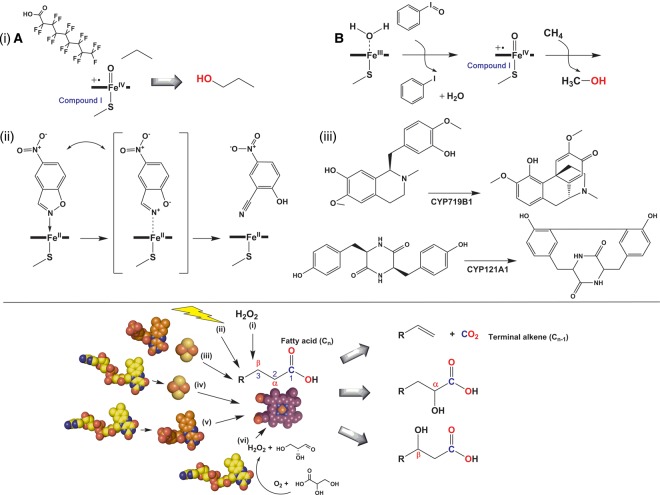
Novel reactions catalyzed by cytochrome P450 enzymes (**A**) and routes to driving catalysis in peroxygenase P450 enzymes (**B**). (**A**) A series of unusual reactions catalyzed by cytochrome P450 enzymes. (i) The hydroxylation of short chain alkanes using (a) the high-activity P450–CPR fusion enzyme P450 BM3 (CYP102A1) from *Bacillus megaterium* to produce propanol from propane in the presence of the ‘decoy’ molecule perfluoro-nonanoic acid (where the decoy molecule acts as a ‘dummy’ substrate to activate the enzyme and to facilitate the binding and oxidation of propane bound in the P450 active site) [24,25] and (b) using wild-type and evolved forms of the CYP153A6 enzyme from *Mycobacterium* sp. HXN-1500 and employing iodosylbenzene to drive oxidation of methane to methanol in small amounts [26–28]; (ii) a Kemp elimination reaction involving the conversion of 5-nitro-benzisoxazole into 2-cyano-4-nitrophenol, in which wild-type and mutant forms of P450 BM3 catalyze the reaction by a redox mechanism predicted [by QM–MM (quantum mechanics–molecular mechanics simulations)] to involve a reaction in which the isoxazole nitrogen becomes co-ordinated to the heme iron [29] and (iii) C–C bond coupling reactions in the formation of salutaridine from *R*-reticuline by CYP179B1 in the morphine synthesis pathway [30] and in the production of the *Mycobacterium tuberculosis* secondary metabolite mycocyclosin from the cyclic dipeptide cyclo-l-Tyr-l-Tyr by *M. tuberculosis* CYP121A1 [31,32]. The schematic in (**B**) shows different methods for driving the catalytic function of P450 peroxygenase enzymes, using the peroxygenase heme prosthetic group (in purple) to transform fatty acids to terminal alkenes and α- and β-hydroxylated fatty acids. (i) Direct formation of P450 compound 0 by reaction with H_2_O_2_. (ii) Formation of H_2_O_2_ using light-mediated excitation of flavin cofactors in the presence of EDTA as an electron donor [33]. NAD(P)H-dependent electron transfer to the P450 using (iii) a phthalate dioxygenase-type enzyme (FMN and 2Fe–2S cluster-binding) [34], (iv) the FAD-binding putidaredoxin reductase and its 2Fe–2S cluster-binding putidaredoxin partner from *P. putida* [35] and (v) the *E. coli* flavodoxin reductase (FAD-binding) and flavodoxin (FMN-binding) to reduce the P450 partner [36]. (vi) The FAD-binding alditol oxidase which produces H_2_O_2_ for peroxygenase catalysis when provided with glycerol or an alternative alditol substrate [37].

The P450s typically act as monooxygenases, binding dioxygen to their ferrous heme iron and ultimately inserting an atom of oxygen into the substrate, with the other oxygen atom being reduced to water [11]. Several different reaction outcomes are feasible according to the particular P450s involved and the type of substrate bound. These include reactions such as hydroxylation and epoxidation (e.g. of saturated and unsaturated fatty acids); N- and S-oxidation reactions; oxidative demethylation, dealkylation and deamination; oxidative and reductive dehalogenation; oxidative C–C bond cleavage, isomerization, aromatic hydroxylation and oxidation of alcohols and aldehydes [12]. More recently discovered P450 reactions (both natural and those resulting from engineered P450 proteins) are shown in Figure 1A.

## P450 redox partners, the peroxide shunt and its applications in cytochrome P450 catalysis

**Figure 2.**
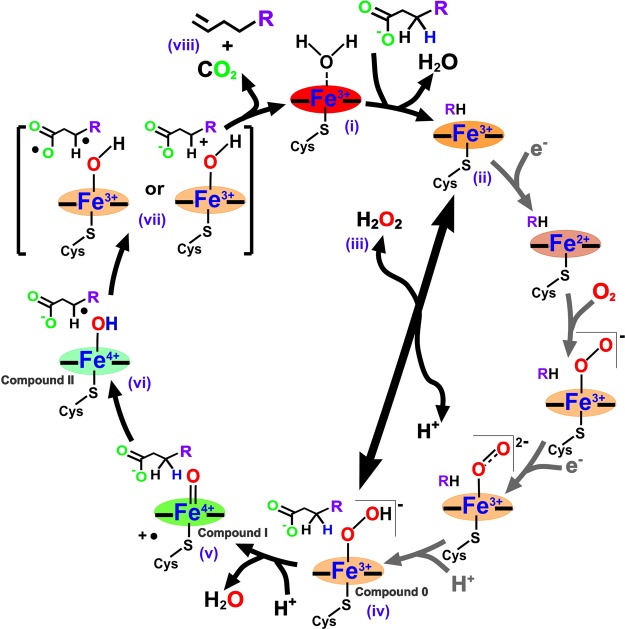
The cytochrome P450 catalytic cycle, incorporating steps specific to peroxygenase activity and the formation of terminal alkenes. (i) The P450 is in a low-spin ferric resting form with its heme iron axially co-ordinated by cysteine thiolate and a H_2_O molecule. (ii) Substrate (fatty acid) binding displaces the axial water and converts the heme iron from low-spin to a high-spin form. (iii) Peroxygenase P450s use the peroxide shunt pathway that bypasses the need for external electrons, and interaction with H_2_O_2_ facilitates direct conversion of the substrate-bound, high-spin P450 to the ferric-hydroperoxo species (compound 0) (iv). Subsequent protonation and dehydration steps yield the reactive ferryl (Fe^IV^)-oxo porphyrin radical cation (compound I) (v), as observed in rapid mixing studies of OleT with perdeuterated arachidic acid substrate [40]. (vi) Compound I abstracts a hydrogen atom from the C_β_ position, resulting in formation of a substrate radical and the ferryl (Fe^IV^)-hydroxo species (compound II) [41]. (vii) Decarboxylation of the fatty acid occurs through subsequent one-electron oxidation of the substrate by compound II to generate a substrate diradical species or a carbocation. (viii) The reactive species can readily eliminate CO_2_, with formation of the C_α_–C_β_ double bond and generation of a terminal alkene. The simultaneous recruitment of a proton to the heme restores the resting, distal water-ligated state of the P450. There is competition between the ‘radical rebound’ reaction in which (a) compound II transfers a hydroxyl radical to the substrate radical to form a hydroxylated fatty acid and the alkene-producing reaction in which (b) compound II abstracts a substrate electron to initiate fatty acid decarboxylation. The decarboxylase reaction dominates in OleT for longer chain substrates, although significant levels of hydroxylation are seen with short chain fatty acids. The P450 heme (illustrated by the iron in the relevant oxidation state and equatorial/axial bonds) is shaded to approximate the color of the relevant heme intermediate species.

The vast majority of P450s requires NAD(P)H-driven redox partner systems for function *in vivo*. In eukaryotes, the microsomal P450s (associated with the endoplasmic reticulum) are tethered to the membrane through an N-terminal transmembrane helical segment, as is their natural redox partner cytochrome P450 reductase (CPR, sometimes referred to as POR, P450 oxidoreductase) [13]. CPR is a diflavin enzyme with two major domains [FAD/NADP(H)-binding and FMN-binding]. NADPH delivers electrons (as a hydride ion) to the FAD domain to reduce it to the hydroquinone state. These two electrons are then transferred sequentially to the flavodoxin-like FMN domain, which in turn shuttles them one at a time to the P450 heme iron to reduce it first to the ferrous state (enabling oxygen binding) and then further reducing the ferric-superoxo species formed to generate a reactive ferric-peroxo state. In subsequent stages in the catalytic cycle, this species undergoes two consecutive protonation steps — forming first a ferric-hydroperoxo species (compound 0) and then undergoing dehydration to produce a ferryl-oxo heme radical cation species (compound I) which is crucial to oxygen insertion chemistry catalyzed by the P450s [14] (Figure 2). Mitochondrial P450s and most P450s from bacteria and archaea use a different redox partner system in which two electrons from NAD(P)H are transferred first to an FAD-binding ferredoxin reductase and then consecutively to an iron–sulfur cluster-binding protein (a ferredoxin, a single electron carrier protein), which in turn delivers two electrons in single steps to the P450 and catalysis ensues as described for the CPR-driven system. In mitochondria, the reductase enzyme is adrenodoxin reductase, which passes electrons to the 2Fe–2S adrenodoxin [15]. Bacterial P450 systems that can use ferredoxins binding 3Fe–4S or 4Fe–4S cofactors have also been reported [16,17].

An alternative route to driving P450 catalysis involves using hydrogen peroxide (H_2_O_2_) or organic surrogates (including cumene hydroperoxide or iodosylbenzene) that can also react with the P450 to generate catalytically competent heme iron-oxo species [e.g. 18]. While these methods are typically not highly efficient and can lead to heme modification and enzyme inactivation, there are also many examples where they have been used effectively as surrogates for the natural redox partner-driven systems. For example, human CYP1A2 was shown to use H_2_O_2_ efficiently to oxidize a heterocyclic aromatic amine, while human CYP3A4 and CYP2D6 could convert the cyano group of the vasodilator drug pinacidil into the corresponding amide using H_2_O_2_ [19,20]. However, this ‘peroxide shunt’ method is rarely used in situations where large amounts of oxidized products are sought, primarily due to the potential for oxidative modification of the heme prosthetic group and active site amino acids, leading to low product formation and inadequate product yields.

## Discovery of the P450 peroxygenases as H_2_O_2_-utilizing P450 enzymes

A breakthrough in the field came with the identification of novel bacterial P450 enzymes involved in fatty acid oxidation reactions. The P450s SP_α_ (CYP152B1 from *Sphingomonas paucimobilis*) and BS_β_ (CYP152A1 from *Bacillus subtilis*) were shown to be active in fatty acid hydroxylation in reactions driven by H_2_O_2_ and to produce α-hydroxylated (2-OH fatty acids) and predominantly β-hydroxylated (∼60% 3-OH fatty acids and ∼40% 2-OH fatty acids), respectively [21,22]. The crystal structure of the CYP152A1 enzyme revealed that the fatty acid substrate binds with its carboxylate group close to the distal face of the heme in the active site pocket and with the carboxylate group making a bidentate interaction with the guanidinium group of an active site arginine residue [23]. In the majority of P450 enzymes, an acid–alcohol amino acid pair (e.g. Asp251 and Thr252 in the *Pseudomonas putida* camphor hydroxylase P450cam [CYP101A1]) is used to relay protons onto iron-oxo species to progress the catalytic cycle. However, these amino acids are replaced by the carboxylate-binding Arg242 and Pro243 in BS_β_ in a conserved region of the peroxygenase enzymes [23]. This pattern recurs in other peroxygenases, including SP_α_ (Arg241 and Pro242) and the *Jeotgalicoccus* sp. OleT (CYP152L1 from *Jeotgalicoccus* sp. ATCC 8456) enzyme (Arg245 and Pro246), pointing to a different evolutionary route taken in these enzymes that accommodates H_2_O_2_-driven catalysis [38,39]. Other notable sequence differences occur in the heme-binding region close to the cysteine thiolate ligand to the heme iron. For example, a strongly conserved phenylalanine residue (e.g. Phe393 in P450 BM3 and Phe350 in P450cam), typically located seven amino acids before the cysteine ligand in the heme-binding loop region, is absent from the peroxygenases. The P450 BM3 F393A/H mutants exhibited substantial increases in their heme Fe^III^/Fe^II^ reduction potential at pH 7.0 compared with the wild-type P450 in both their substrate-free (redox potential increases of 115/95 mV, respectively) and arachidonic acid-bound forms (redox potential increases of 138/113 mV, respectively) [42]. In studies of the OleT peroxygenase, the heme iron reduction potential was shown to be very positive: −103 mV (substrate-free) and −105 mV (arachidic acid [C20:0]-bound) vs. the normal hydrogen electrode (NHE) at pH 7.0. The similarities in the OleT redox potentials in substrate-bound/-free forms may, in part, reflect the influence of the substrate and its carboxylate group on the heme environment, but the overall very positive potential of the OleT heme iron is likely also due, in part, to the altered structural organization at the heme proximal face [39].

Girhard et al. characterized an ortholog of P450 BS_β_ from *Clostridium acetobutylicum* (P450_CLA_, CYP152A2) with ∼57% amino acid sequence identity. P450_CLA_ catalyzed H_2_O_2_-dependent hydroxylation of various fatty acids with both α- and β-hydroxylated fatty acids produced. The enzyme was also shown to be functional with redox partner systems (*Escherichia coli* flavodoxin reductase/flavodoxin [FLDR/FLD] or the P450 BM3 reductase domain with NADPH reductant), and over extended reaction times could produce considerably larger amounts of product compared with shorter reactions using 200 μM H_2_O_2_ to drive catalysis [36]. In subsequent studies, Girhard et al. [33] also demonstrated a light-driven method for peroxygenase activation, using light-mediated excitation of flavin cofactors with EDTA as an electron donor to produce H_2_O_2_ in the reaction vessel for P450_CLA_-dependent oxidation of myristic acid (C14:0) substrate. Thus, alternative and potentially more productive routes to peroxygenase-mediated catalysis exist in addition to H_2_O_2_-mediated oxidation reactions.

## OleT and its characterization as a fatty acid decarboxylating enzyme

In a key study by Rude et al. [43], many peroxygenase enzymes were identified and characterized for their ability to generate oxidized products from fatty acids. The authors identified a *Jeotgalicoccus* sp. (ATCC 8456) as a bacterium that produced several terminal olefins, with 18-methyl-1-nonadecene being the most abundant. Terminal olefin production was also demonstrated in other *Jeotgalicoccus* spp. The enzyme was isolated from *Jeotgalicoccus* sp. ATCC 8456 and expressed in *E. coli*, leading to the identification of terminal olefin products including 1-pentadecene and 1-heptadecene. The protein sequence was characterized as that of a peroxygenase-type cytochrome P450 and formally classified as CYP152L1, with the trivial title OleT due to its olefin-forming capability [39,43]. Thereafter, *in vitro* studies were done to demonstrate that the respective *n* − 1 terminal alkenes were produced from C14:0, C16:0, C18:0 and C20: fatty acids, alongside smaller amounts of α- and β-hydroxylated fatty acids. These findings led Rude et al. to analyze H_2_O_2_-dependent product formation from many homologous CYP152 family P450s in their reaction with palmitic acid (C16:0). These data revealed the formation of 1-pentadecene in CYP152 enzymes from *Corynebacterium efficiens*, *Kocuria rhizophila*, *Methylobacterium populi* and *B. subtilis*. In the case of the *B. subtilis* BS_β_ enzyme, the alkene product comprised ∼15% of the total product alongside α- and β-hydroxylated fatty acids, suggesting that selected CYP152 family enzymes could either hydroxylate or decarboxylate their fatty acid substrates using distinct mechanisms. These findings stimulated further research into the potential of OleT and other peroxygenases for alkene production in the area of biofuels and fine chemicals [43].

**Figure 3.**
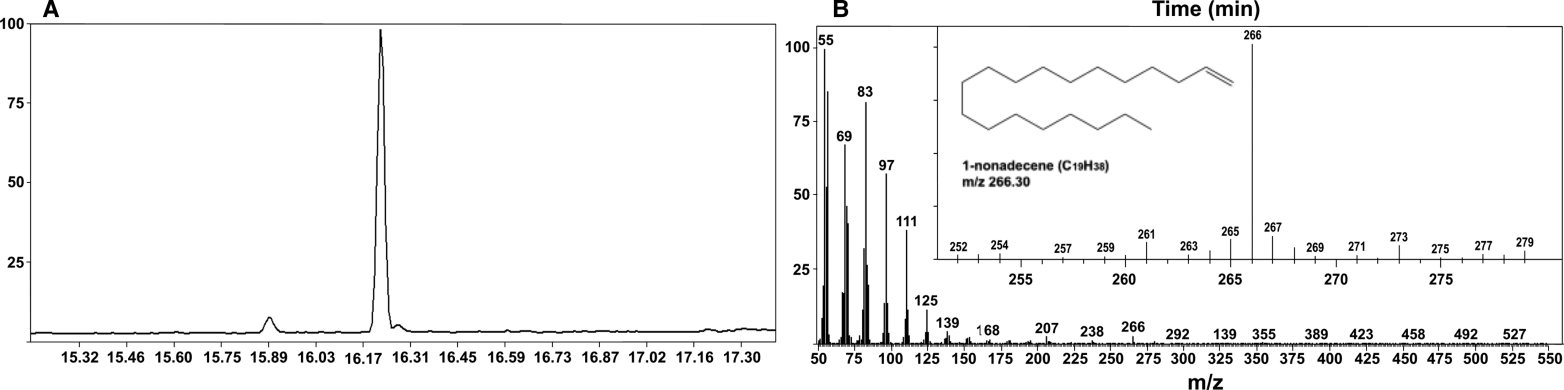
Oxidative decarboxylation of arachidic acid by OleT. (**A**) The total ion count from gas chromatography separation of the C19 terminal alkene 1-nonadecene in the reaction of OleT with arachidic acid (C20:0) and with a major peak at 16.21 min. (**B**) Mass spectrometric analysis of the peak (16.21 min) with the inset highlighting the region of the mass ion with *m*/*z* 266, consistent with the formation of the 1-nonadecene product. The identification of the 1-nonadecene product was confirmed using tandem (MS–MS) mass spectrometry.

Further studies on OleT revealed that the P450 could bind saturated fatty acids from ∼C10:0 to C20:0 with moderate to high affinity (*K*_d_ = 0.29 μM in the case of C20:0), as determined by fatty acid-induced shifts in the heme spectrum that report on the conversion of low-spin heme iron toward the high-spin state on substrate association. Shorter chain fatty acids induced less extensive high-spin shifts [39]. Figure 3 shows separation (by gas chromatography) and mass spectrometry characterization of the C19 alkene nonadecene, formed by OleT-catalyzed decarboxylation of the C20:0 substrate arachidic acid. In other research exploring alternative routes to driving peroxygenase catalysis, Liu et al. [34] demonstrated that OleT activity could be supported by NAD(P)H-dependent redox partners: using either an OleT-phthalate dioxygenase reductase [PDOR]-like fusion enzyme (with the reductase domain of the *Rhodococcus* sp. CYP116A1 enzyme) or the *E. coli* flavodoxin reductase and flavodoxin proteins (FLDR and FLD). Dennig et al. also demonstrated that several short chain fatty acids (C9:0 to C4:0) were substrates for OleT, despite many of these failing to give significant high-spin optical binding shifts. Substantial conversion of these substrates to their respective terminal alkenes (and hydroxylated products) was achieved using the *P. putida* P450cam CamA/B redox system (putidaredoxin reductase/putidaredoxin) when applying either formate dehydrogenase (FDH)-dependent or phosphite dehydrogenase-dependent NAD(P)H regeneration systems. In their *in vitro* system, Dennig et al. [35] achieved best alkene production using stearic (C18:0) acid, with a product titer of 0.93 g l^−1^ and a total turnover number of >2000 at low catalyst loading levels. These findings likely indicate that the bacterial peroxygenase P450s retain a viable redox partner (e.g. ferredoxin) binding site on the proximal face of the protein, even though the H_2_O_2_-driven catalytic route is likely to be the physiologically relevant one. This retention of OleT catalytic function with diverse redox partners clearly presents opportunities to optimize alkene production through identifying the most efficient redox partner systems.

## The structure and catalytic mechanism of OleT as a model for the P450 peroxygenases

**Figure 4.**
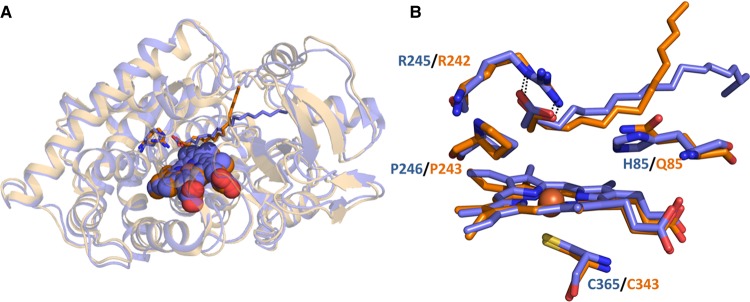
A structural comparison of the peroxygenase P450s OleT and BS_β_. (**A**) Crystal structure of the OleT P450 (blue) with arachidic acid bound (PDB 4L40) and of the BS_β_ P450 (orange) bound to palmitic acid (PDB 1IZO). The hemes are shown in spheres with the conserved arginine residues (Arg245 in OleT and Arg243 in BS_β_, that interact with the substrate carboxylate group) shown in sticks and colored to match their relative structures. The two substrate molecules are shown to occupy different entry/exit channels as they approach the surface of the protein structures. (**B**) Close up of the active site of the OleT and BS_β_ P450s, showing the conserved active site Arg and Pro residues on the I-helix, as well as His 85 (OleT) and Gln 85 (BS_β_) close to the substrates. The interactions between these arginine residues and their respective substrate carboxylate groups facilitate H_2_O_2_ activation by use of the substrate carboxylate as a general acid to assist in the formation of the catalytic ferryl (Fe^IV^)-oxo species.

The crystal structure of OleT was obtained in both substrate-free and arachidic acid-bound forms at 2.3 and 2.5 Å resolution, respectively. The overall OleT structure is not altered substantially between substrate-free and -bound forms, and also closely resembles that of P450 BS_β_, with a root mean square deviation of 0.99 Å for 379 Cα atoms [23,39]. Despite this close similarity, there are structural differences observed which apparently explain the ability of OleT to catalyze the oxidation of longer chain substrates by comparison with P450 BS_β_. These are found in the flexible FG-loop region (between the F- and G-helices) and in the nearby C-terminal loop sections, which are both regions that line the OleT-binding pocket and are close to the fatty acid terminal methyl group. Leu177 in the FG-loop closes a narrow solvent access channel (that remains open in P450s SP_α_ and BS_β_), while the substrate-binding pocket is extended in OleT compared with BS_β_ due to mutations in the N-terminal β-sheet section. These changes allow OleT to accommodate the C20:0 arachidic acid, which near-completely fills the active site cavity [39] (Figure 4).

**Figure 5.**
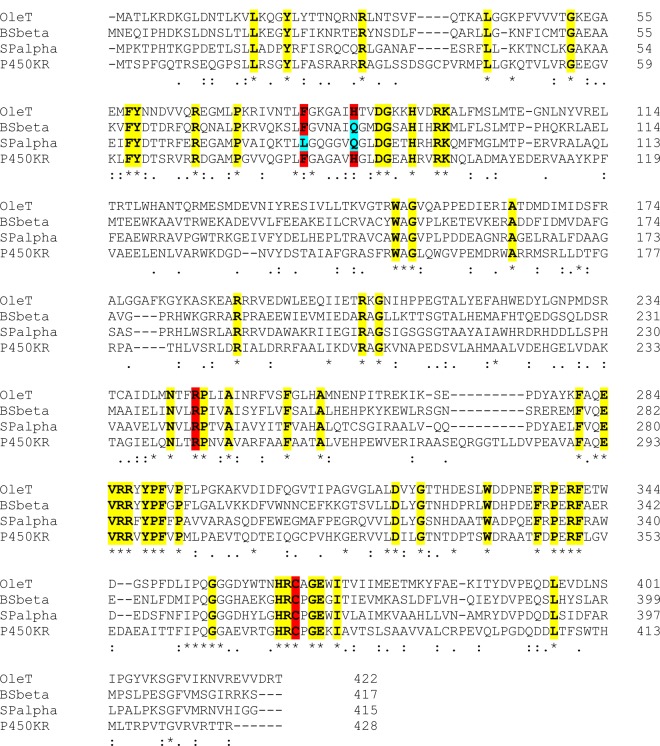
Amino acid alignment of selected P450 peroxygenase enzymes. The amino acid sequences of the P450s OleT (CYP152L1 from *Jeotgalicoccus* sp. 8456), BS_β_ (CYP152A1 from *Bacillus subtilis*), SP_α_ (CYP152B1 from *S. paucimobilis*) and KR (CYP152T1 from *Kocuria rhizophila*) are aligned. Residues in red/cyan are located close to the fatty acid carboxylate group in the active site. The Arg245 guanidinium group makes a bidentate interaction with the substrate carboxylate in OleT, with the substrate C_α_ and C_β_ atoms located at 5.1 and 5.7 Å from the heme iron, respectively. A water molecule is located between His85 and the carboxylate, and the His85 imidazole group is directed toward the heme iron at a distance of 5.8 Å. The imidazole is positioned between the heme edge and Phe79, and has no polar interactions with other residues. SP_α_ and BS_β_ have residues Gln84 and Gln85, respectively, rather than the histidines found in OleT (His85) and KR (His 89). SP_α_ does not undergo significant substrate binding-induced high-spin heme iron accumulation, and BS_β_ gives only small heme spectral shifts [46]. The OleT H85Q mutant behaves similarly to BS_β_ with less extensive substrate-dependent type I spin-state shifts, while OleT Phe79 mutants diminish substrate affinity [44]. OleT Arg245 is conserved in all peroxygenase sequences identified to date and is pivotal in substrate recognition and catalysis. The conserved sets of amino acid residues highlighted in yellow are those most extensively retained across the peroxygenases, while the cysteine thiolate proximal ligand to the heme iron (Cys365 in OleT, colored red in each peroxygenase) is also completely conserved. However, the structure of the cysteine-containing binding loop on the proximal face of the heme is different from those found in typical monooxygenase P450s and is where electron delivery occurs from redox partners that bind to this region. This observation suggests an evolutionary route taken toward H_2_O_2_-dependent activity, and OleT and other bacterial peroxygenases have Arg-Pro motifs (Arg245–Pro246) that replace an acid–alcohol amino acid pair (Asp251–Thr252 in P450cam) in the active site region that is considered crucial for protonation of heme iron-oxo species in the canonical P450 monooxygenase catalytic cycle. The Arg-Pro motif provides a fatty acid carboxylate-binding site in the peroxygenases, while direct interaction of H_2_O_2_ with the substrate-bound heme bypasses the necessity for oxygen binding, reduction and protonation steps used in typical monooxygenase P450s, and leads directly to the formation of a reactive ferric-hydroperoxo (compound 0) species.

In the arachidic acid-bound OleT, the carboxylate group interacts with Arg245, while a nearby histidine (His85) was proposed as a potential proton donor to the reactive P450 compound I (ferryl-oxo heme radical cation) intermediate that is a key species in substrate oxidation in almost all P450s. However, His85 appears as a glutamine in both the SP_α_ and BS_β_ enzymes. Rude et al. [43] reported that a Q85H mutation in P450 BS_β_ did not abolish activity, and instead enhanced the rate of palmitic acid decarboxylation by ∼50%, while increasing production of β-hydroxylated palmitic acid at the expense of the α-hydroxylated product. The OleT H85Q mutant was also generated and resulted in a far smaller conversion to the high-spin state on binding arachidic acid in comparison with wild-type OleT [44]. These properties are similar to those of the wild-type BS_β_ and SP_α_ P450s, where only a minor spectral shift (BS_β_) or no significant shift (SP_α_) occurs on binding substrates [45,46]. These data cast some doubt on a role for OleT His85 as a key proton donor, but simultaneously confirms the importance of the residue at this position in peroxygenase activity and regulation of spin-state equilibrium. Figure 5 shows an amino acid alignment of OleT with other selected peroxygenases, highlighting conserved residues important for substrate binding and catalysis.

In work to probe peroxygenase catalytic mechanism, Makris' group used stopped-flow and spectroscopic approaches to characterize heme iron-oxo species involved in fatty acid hydroxylation and decarboxylation reactions. Transient kinetics methods proved to be a very useful tool for dissecting the chemical mechanism of OleT, including identification of the critical intermediates in the decarboxylation reaction sequence. The C–C scission of a C*_n_* chain length fatty acid to produce a C*_n_*_−1_ 1-alkene and CO_2_ co-product requires the abstraction of a hydrogen atom from the C_β_ position and an additional reducing equivalent from the substrate to form the terminal C_α_−C_β_ double bond. In summary, this amounts to removal of a substrate hydride. The nature of this abstraction, combined with structural and functional analogy to the well-studied CYP152 hydroxylases BS_β_ and SP_α_ that exhibit significant steady-state ^2^H kinetic isotope effects (KIE ≥4) [47], strongly implies the intermediacy of the iron(IV)-oxo pi-cation radical species (compound I or CYP-I) as an electrophilic oxidant in OleT. It is notable that a large substrate ^2^H KIE (*k*_H_/*k*_D_ ∼14) was also observed in an unrelated O_2_-dependent CYP that can decarboxylate isovalerate to isobutene [47].

The intrinsic difficulties associated with isolating ferryl intermediates in P450s are well documented and have been covered in a recent review [48]. Although several strategies have been employed in attempts to promote the accumulation of CYP-I, the most common (and successful) way has been through the addition of strong oxidants such as meta-chloroperoxybenzoic acid (mCPBA) to the substrate-free ferric enzyme [14,49]. Considered a ‘shunt pathway’ in O_2_-dependent P450s, this stratagem circumvents rate-limiting electron transfer processes, and in the absence of a bound substrate molecule can sometimes permit the observation of compound I. The obligate requirement of a bound substrate-derived carboxylate moiety for efficient H_2_O_2_ activation in CYP152 enzymes requires a slightly different stratagem to be used. Provided that C–H abstraction is rate-limiting with respect to the binding and rearrangement of H_2_O_2_ to form CYP-I, a sufficiently large substrate ^2^H KIE could be used as a means to trap the fleeting intermediate. Similar strategies have been successfully employed as a means to trap reactive O_2_ intermediates in several non-heme iron-oxygenases [50,51].

**Figure 6.**
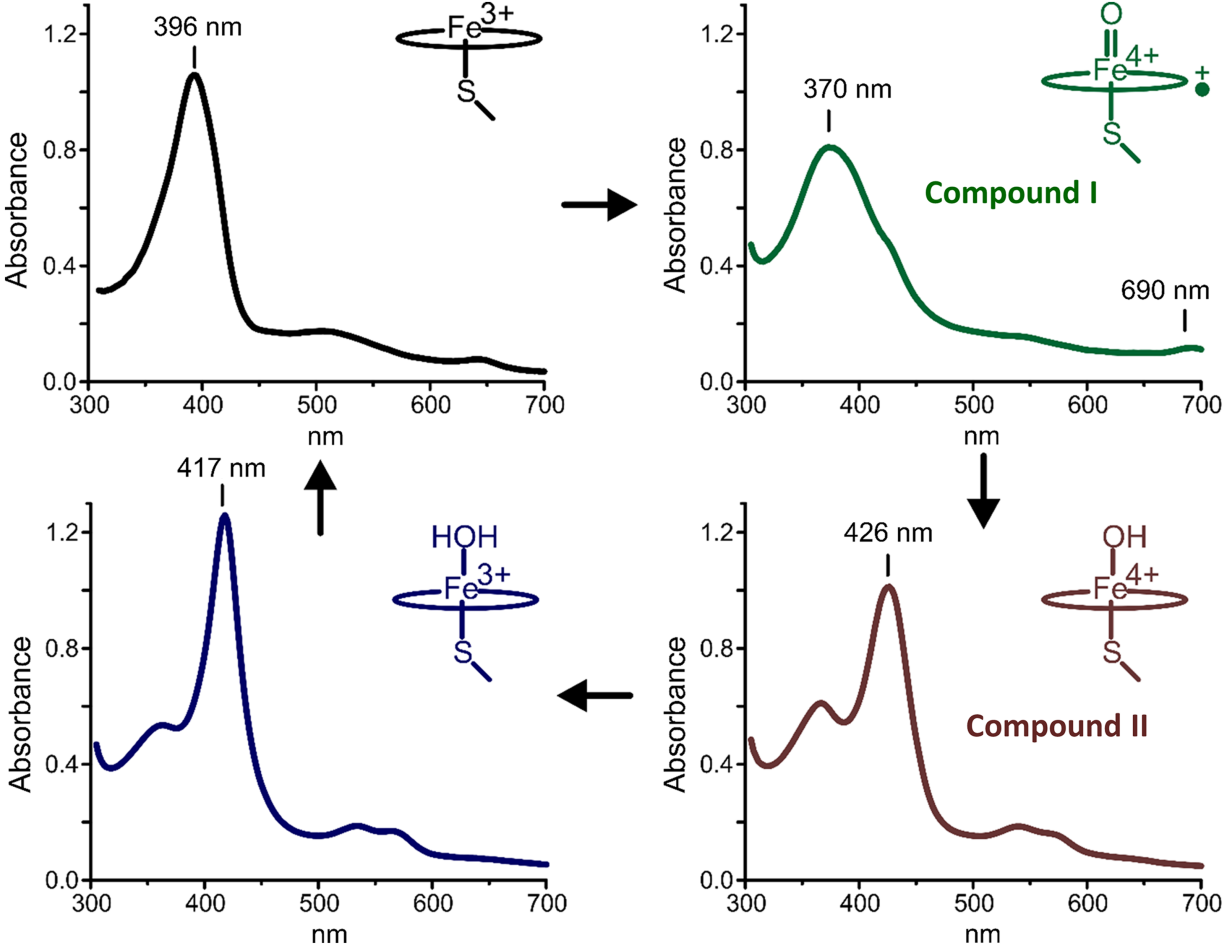
Spectral intermediates in the OleT catalytic cycle. From top left to bottom left in a clockwise direction, the UV–visible spectra for four key P450 catalytic cycle species are shown. **Top left**: the ferric, high-spin form of OleT, as observed for the OleT complex with arachidic acid (C20:0). The Soret peak is at 396 nm, with a small feature at 650 nm indicative of a cysteine thiolate-to-high-spin ferric heme iron charge-transfer species. **Top right**: the spectrum of OleT compound I (Fe^IV^=oxo porphyrin radical cation) with characteristic spectral maxima at ∼370 and 690 nm. **Bottom right**: the OleT compound II (Fe^IV^-hydroxo) spectrum following hydrogen atom abstraction from the substrate and with a Soret peak maximum at 426 nm. **Bottom left**: the spectrum of the resting, low-spin ferric form of OleT following completion of the catalytic cycle and with the Soret maximum at 417 nm.

In a recent study, Grant et al. [40] probed the decarboxylation of perdeuterated arachidic acid (C20:0), a chain length that approximates that of the native OleT substrates [39], in stopped-flow studies. The rapid mixing of an OleT : perdeuterated arachidic acid ternary complex leads to a disappearance of the high-spin ferric enzyme (*λ*_max_ ∼392 nm; Figure 6, top left), and the rapid generation of a new species with a Soret maximum at 370 and an additional absorption band at 690 nm (Figure 6, top right). The features of the OleT intermediate, originally described for chloroperoxidase [52], have now become synonymous with CYP-I species and are common to other thiolate-ligated heme enzymes [14,49,53]. Over time, the Ole-I (OleT compound I) decays to the ferric low-spin enzyme (Figure 6, bottom left). Importantly, this result directly confirmed that CYP-I species could participate in oxidations that are not finalized by an oxygen rebound step, as hypothesized for many atypical CYP reactions that resemble OleT oxidative decarboxylation (e.g. desaturations [54,55]).

**Figure 7.**
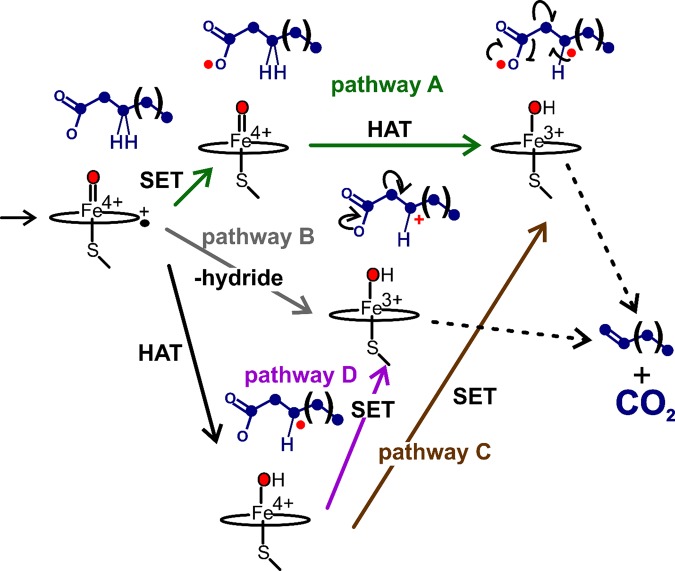
Potential pathways for substrate decarboxylation in OleT and other peroxygenases. The net abstraction of a hydride from a fatty acid substrate could involve HAT from the Cβ and a SET step from the carboxylate (pathway A or C) or from Cβ (pathway D) to afford a substrate diradical species or Cβ carbocation, respectively. Formation of a substrate Cβ carbocation could proceed from the concerted abstraction of a substrate hydride (pathway B). On the basis of a large substrate ^2^H kinetic isotope effect for OleT compound I decay and observation of a compound II-like species in transient kinetic studies, either pathway C or D is most likely operative. Rearrangement of the Cβ carbocation or the diradical results in formation of the terminal olefin and the carbon dioxide co-product.

Despite the successful isolation of Ole-I in the reactions of arachidic acid as well as shorter chain length substrates [40], several interesting questions remained regarding the nature and ordering of the abstraction steps. Abstraction of a substrate hydride affords several intriguing mechanistic possibilities that are briefly summarized in Figure 7. The first of these (pathway A) consists of single electron transfer (SET) from the substrate to compound I followed by hydrogen atom transfer (HAT) to compound II. This proposal can be ruled out from the observation that Ole-I failed to appreciably accumulate in reactions with protiated arachidic acid, implying a significant substrate ^2^H KIE. In an attempt to parse out the remaining possibilities, the decay kinetics of Ole-I were closely tracked in a follow-up study [41]. Careful examination of the photodiode array data revealed an intermediate that kinetically behaves as the direct decay product of Ole-I. The spectral properties of this species (shown in Figure 6, bottom right), with a Soret maximum at 426 nm and hyperporphyrin character, are highly similar to protonated iron(IV)-hydroxide (compound II) species that have been prepared in CYP158 by Green and colleagues and that result from the oxidation of the protein framework [53]. Provided that this intermediate is indeed Ole-II (OleT compound II), one can eliminate a possible mechanism involving concerted hydride transfer by Ole-I (Figure 7, pathway B). The most remarkable feature of this species, however, is that it is observable at all and decays very slowly (∼10 s^−1^) to the final resting state of the enzyme. This result may imply that stabilization of an extremely long-lived substrate radical in OleT is central to preventing oxygen rebound from occurring, and allows for SET, either from the carboxylate or the C_β_ position (Figure 7, pathways C and D), to ensure the decarboxylation outcome. Hsieh and Makris [56] recently adapted the ‘decoy’ approach of Shoji et al. [57] to evaluate OleT radical lifetimes using norcarane and methylphenylcyclopropane radical clock substrates. The effective hydroxylation of these substrates and calibrated product profiles are consistent with short picosecond radical lifetimes that are more in line with those measured for many other CYP hydroxylases. Collectively, these results intimate that co-ordination of the substrate, rather than the electronic structure of the ferryl intermediates, may be the most critical element for maintaining OleT chemoselectivity. Parsing out the precise metrics that effectively negotiate between efficient HAT and oxygen rebound, including the distance of the substrate C–H bond relative to the oxo-unit, may give rise to a better understanding of how P450 oxidations, such as those catalyzed by OleT, can be better leveraged for the synthesis of valuable molecules using a richer panel of substrates.

## Novel products and biotechnological applications

Further studies of OleT revealed that products other than terminal alkenes and α- and β-hydroxylated fatty acids were formed in small amounts. These arose from secondary reactions on primary products, leading to the generation of (i) tridec-1-en-1-ol and (ii) 2-hydroxytetradec-2-enoic acid from the 2-hydroxytetradecanoic acid primary product (and resulting from its (i) decarboxylation and (ii) desaturation at the C_α_–C_β_ bond, respectively) and (iii) 3,4-dihydroxytetradec-2-enoic acid, resulting from the further hydroxylation and desaturation of 3-hydroxytetradecanoic acid. It remains unclear whether these molecules are of relevance to the bacterium's metabolism, or simply reflect that primary products retain sufficient affinity for the OleT active site that secondary hydroxylation or decarboxylation events can occur [37]. OleT was also reported to produce 2-alkanone products in reactions with palmitic (C16:0) and stearic (C18:0) acid [35].

Studies on the SP_α_ and BS_β_ peroxygenases by Watanabe and Shoji's groups demonstrated that very short chain fatty acids (as short as acetic acid, used at high concentration) could be used as ‘decoy substrates’ in order to prime these enzymes for activity and to facilitate the oxidation of other non-carboxylate-containing molecules, including 1-methoxynaphthalene, ethylbenzene and styrene [24,25]. Following from this work, Hsieh and Makris engineered a P246D mutation into the OleT active site and found that this mutation blocked fatty acid access to the heme and prevented any significant production of alkene or hydroxylated fatty acid products. However, several new substrates lacking carboxylate groups were efficiently oxidized, including linear (nonane), cyclic (cyclohexane) and aromatic (styrene) hydrocarbons, demonstrating that the introduced active site glutamate can provide the carboxylate required for effective peroxide utilization [56]. There is clearly potential for further expansion of research in this area with the peroxygenases, although acceptable substrate size may be limited and further protein engineering to enhance substrate access and/or catalytic efficiency would likely be required. Further studies should also investigate large-scale fermentation of OleT in *E. coli* (or other bacterial expression cells) in order to optimize OleT expression and production of alkenes. The co-expression of efficient redox partners for OleT may provide the best route for alkene production, with the study of Dennig et al. [35] showing that the CamA/B enzymes are effective redox partners for OleT. Alkenes produced in this way could have multiple applications in chemical processes, including in the production of polymers/plastics, hydrogen halides amines and aldehydes (by hydroamination and hydroformylation, respectively), and ketones using ozonolysis.

The OleT heme iron Fe^III^/Fe^II^ redox potentials in substrate-free (−103 mV) and arachidic acid-bound (−105 mV) forms of OleT (vs. NHE at pH 7.0) are substantially more positive than those typical of other (non-peroxygenase) bacterial P450s. Our preliminary data for the P450 KR peroxygenase (CYP152T1) from *K. rhizophila* also indicate very positive potentials for substrate-free and fatty acid-bound forms of this P450 ([39]; Tee, K.-L., McLean, K.J., Leys, D. and Munro, A.W., unpublished data). These positive heme iron potentials may explain in part why redox partner systems, such as CamA/B, are capable of supporting OleT catalysis, on the grounds that there is a thermodynamically very favorable route of electron transfer [from NAD(P)H at −320 mV vs. NHE at pH 7.0] to the P450 heme iron in these peroxygenases. However, the extent to which electron transfer is coupled to fatty acid substrate oxidation remains to be established in this type of redox system. In contrast, comparable Fe^III^/Fe^II^ redox potentials for the well-studied P450 BM3 (CYP102A1) and P450cam in their substrate-free and substrate-bound forms are considerably more negative at −368 ± 6 mV (substrate-free) and −239 ± 6 mV (arachidonic acid-bound), and at −300 mV (substrate-free) and −170 mV (*D*-camphor-bound), respectively, vs. NHE at pH 7.0 [58,59].

## Summary

The bacterial P450 peroxygenases have attracted a great deal of scientific interest following the discovery that many of these enzymes are able to oxidatively decarboxylate fatty acids to form terminal alkene products with potential applications in biofuel production and for general fine chemical uses [43]. Arguably more importantly, the catalytic mechanism of the OleT (CYP152L1) peroxygenase has been interrogated using rapid reaction methods and spectroscopic techniques, enabling the identification and characterization of the transient, reactive iron-oxo species compound I (ferryl-oxo porphyrin radical cation) and compound II (ferryl-hydroxo), that were shown to catalyze fatty acid monooxygenation (to form hydroxylated fatty acids) and decarboxylation (to form *n* − 1 terminal alkenes), respectively [14,40,41]. In combination with the detailed structural characterization of OleT (and other peroxygenase P450s), the analysis of peroxygenase product profiles, discovery of alternative routes to driving peroxygenase catalysis and strategies that facilitate their oxidation of non-fatty acid substrates, the scene is now clearly set for the wider applications of P450 peroxygenases in synthetic biology [25,33–35,39,44,57].

## Abbreviations

BS_β_: P450 BS_β_, CYP152A1 from *Bacillus subtilis*
CamA/B: putidaredoxin reductase/putidaredoxin from *Pseudomonas putida*
CO: carbon monoxide
CPR: cytochrome P450 reductase
CYP: cytochrome P450 monooxygenase
CYP-I: cytochrome P450 compound I (Fe^IV^-oxo pi-cation radical)
FLD: flavodoxin from *Escherichia coli*
FLDR: flavodoxin reductase from *Escherichia coli*
H_2_O_2_: hydrogen peroxide
HAT: hydrogen atom transfer
KIE: kinetic isotope effect
KR: P450 KR, CYP152T1 from *Kocuria rhizophila*
NHE: normal hydrogen electrode
Ole-I: OleT compound I
OleT: CYP152L1 from *Jeotgalicoccus* sp. ATCC 8456
P450: cytochrome P450 monooxygenase
P450_CLA_: CYP152A2 from *Clostridium acetobutylicum*
SET: single electron transfer
SP_α_: P450 SP_α_, CYP152B1 from *Sphingomonas paucimobilis*.
